# Progression of Hypertrophy and Myocardial Fibrosis in Aortic Stenosis: A Multicenter Cardiac Magnetic Resonance Study

**DOI:** 10.1161/CIRCIMAGING.117.007451

**Published:** 2018-06-18

**Authors:** Russell J. Everett, Lionel Tastet, Marie-Annick Clavel, Calvin W.L. Chin, Romain Capoulade, Vassilios S. Vassiliou, Jacek Kwiecinski, Miquel Gomez, Edwin J.R. van Beek, Audrey C. White, Sanjay K. Prasad, Eric Larose, Christopher Tuck, Scott Semple, David E. Newby, Philippe Pibarot, Marc R. Dweck

**Affiliations:** 1 British Heart Foundation Centre for Cardiovascular Science (R.J.E., J.K., M.G., E.J.R.v.B., C.W., S.S., D.E.N., M.R.D.); 2 Edinburgh Imaging Queen’s Medical Research Institute Facility (E.J.R.v.B., S.S.); 3 Edinburgh Clinical Trials Unit, Usher Institute of Population Health Sciences and Informatics (C.T.); 4 University of Edinburgh, United Kingdom. Department of Medicine, Quebec Heart and Lung Institute, Canada (L.T., M.-A.C., R.C., E.L., P.P.).; 5 Department of Cardiovascular Science, National Heart Center Singapore (C.W.L.C.).; 6 Cardiovascular Magnetic Resonance Unit, Royal Brompton Hospital, London, United Kingdom (V.S.V., S.K.P.).; 7 Norwich Medical School, Norfolk and Norwich University Hospital, United Kingdom (V.S.V.).; 8 First Department of Cardiology, Poznan University of Medical Sciences, Poland (J.K.).; 9 Hospital del Mar Medical Research Institute, Universitat Pompeu Fabra, Barcelona, Spain (M.G.).

**Keywords:** aortic valve stenosis, fibrosis, gadolinium, hypertrophy, magnetic resonance imaging

## Abstract

**Background:**

Aortic stenosis is accompanied by progressive left ventricular hypertrophy and fibrosis. We investigated the natural history of these processes in asymptomatic patients and their potential reversal post-aortic valve replacement (AVR).

**Methods:**

Asymptomatic and symptomatic patients with aortic stenosis underwent repeat echocardiography and magnetic resonance imaging. Changes in peak aortic-jet velocity, left ventricular mass index, diffuse fibrosis (indexed extracellular volume), and replacement fibrosis (late gadolinium enhancement [LGE]) were quantified.

**Results:**

In 61 asymptomatic patients (43% mild, 34% moderate, and 23% severe aortic stenosis), significant increases in peak aortic-jet velocity, left ventricular mass index, indexed extracellular volume, and LGE mass were observed after 2.1±0.7 years, with the most rapid progression observed in patients with most severe stenosis. Patients with baseline midwall LGE (n=16 [26%]; LGE mass, 2.5 g [0.8–4.8 g]) demonstrated particularly rapid increases in scar burden (78% [50%–158%] increase in LGE mass per year). In 38 symptomatic patients (age, 66±8 years; 76% men) who underwent AVR, there was a 19% (11%–25%) reduction in left ventricular mass index (*P*<0.0001) and an 11% (4%–16%) reduction in indexed extracellular volume (*P*=0.003) 0.9±0.3 years after surgery. By contrast midwall LGE (n=10 [26%]; mass, 3.3 g [2.6–8.0 g]) did not change post-AVR (n=10; 3.5 g [2.1–8.0 g]; *P*=0.23), with no evidence of regression even out to 2 years.

**Conclusions:**

In patients with aortic stenosis, cellular hypertrophy and diffuse fibrosis progress in a rapid and balanced manner but are reversible after AVR. Once established, midwall LGE also accumulates rapidly but is irreversible post valve replacement. Given its adverse long-term prognosis, prompt AVR when midwall LGE is first identified may improve clinical outcomes.

**Clinical Trial Registration:**

URL: https://www.clinicaltrials.gov. Unique identifiers: NCT01755936 and NCT01679431.

## Abstract 2

Supplemental Digital Content is available in the text.

**See Editorial by Treibel et al**

**CLINICAL PERSPECTIVE**

Left ventricular hypertrophy and myocardial fibrosis are key processes in aortic stenosis that can be assessed by cardiovascular magnetic resonance. However, longitudinal changes in myocardial hypertrophy and fibrosis before and after aortic valve replacement are not well studied. We performed a multicenter prospective cohort study of 99 subjects who underwent serial echocardiography and cardiovascular magnetic resonance with assessment of left ventricular mass, diffuse fibrosis (T1 mapping), and replacement fibrosis (late gadolinium enhancement). Sixty-one subjects were asymptomatic allowing us to assess the natural history of hypertrophy and fibrosis for 2.1±0.7 years. Thirty-eight symptomatic subjects underwent aortic valve replacement with repeat imaging after 1 year allowing us to assess the left ventricular remodeling response to surgery. Our data demonstrate that in patients with aortic stenosis, cellular hypertrophy and diffuse interstitial fibrosis increase in a balanced and exponential manner before reversing (at different rates) after aortic valve replacement. Midwall replacement fibrosis also accumulates rapidly once established in the ventricle but crucially seems irreversible after aortic valve replacement. The myocardial scar burden that patients develop while waiting for surgery, therefore, persists into the long term. This is an important observation because midwall fibrosis has consistently demonstrated an association with adverse outcome in a proportionate manner across multiple patient cohorts. Our data, therefore, suggest that prompt valve replacement as soon as midwall fibrosis develops may hold promise in improving clinical outcomes in patients with aortic stenosis, and this hypothesis will be examined in the currently-recruiting EVOLVED trial (Early Valve Replacement guided by Biomarkers of Left Ventricular Decompensation in Asymptomatic Patients with Severe Aortic Stenosis).

Aortic stenosis (AS) is the most common valve disease requiring operative intervention in high-income countries.^1^ Traditional assessments of AS severity focus on the degree of hemodynamic obstruction in the valve. However, the importance of the myocardial response to pressure overload has been increasingly appreciated, especially when considering the development of symptoms and long-term prognosis after valve intervention.^2^ Left ventricular hypertrophy (LVH) initially normalizes wall stress and maintains cardiac output for many years, if not decades. However, with time, the left ventricle (LV) decompensates and the patient transitions toward heart failure, symptoms, and adverse events.

Pathological studies have suggested that this transition from hypertrophy to heart failure is driven by a combination of myocyte cell death and myocardial fibrosis.^3^ Magnetic resonance imaging (MRI) can detect focal myocardial fibrosis using late gadolinium enhancement (LGE) and estimates diffuse interstitial fibrosis with T1 mapping. A midwall pattern of LGE observed in AS acts as a marker of LV decompensation and is associated with an adverse prognosis after surgery.^4–8^ However, to date, we have lacked longitudinal studies to assess how LVH and fibrosis progress with time and how aortic valve replacement (AVR) affects these processes. The aims of this prospective multicenter study were to assess the time course of LVH and fibrosis in patients with asymptomatic AS and to determine how they are affected in symptomatic patients who undergo AVR.

## Methods

### Study Population

Patients were recruited from 2 large prospective observational MRI studies investigating the natural history of AS (NCT01755936, Edinburgh Heart Centre, United Kingdom,^7^ and NCT01679431, Quebec Heart and Lung Institute, Canada^9^). In both studies, patients underwent comprehensive clinical and echocardiographic assessment including repeat MRI. Eligible participants had undergone at least 2 serial MRI scans. Symptomatic patients had AVR shortly after baseline MRI allowing us to assess the reverse remodeling effect of surgery on repeat scans. The study was conducted in accordance with the Declaration of Helsinki and approved by the local research committees. Written informed consent was obtained from all participants. Study data can be made available to other researchers on request to the corresponding author.

### Echocardiography

Comprehensive transthoracic echocardiography was performed in all patients to assess AS severity as per clinical guidelines (Data Supplement).

### Cardiac Magnetic Resonance

MRI was performed using both 1.5T and 3T scanners, and standard cine images of the LV were acquired. LGE was performed 15 minutes after administration of gadobutrol. T1 mapping was performed using the Modified Look-Locker Inversion-recovery sequence^10^ before and 15 to 20 minutes after gadolinium contrast administration. Although there was variation in the scanners used at the different centers, all patients underwent standardized baseline and repeat imaging within their respective institutions (Data Supplement). To account for potential interscanner variation in T1 measurements,^11^ extracellular volume (ECV)–derived T1 mapping measures were obtained to normalize myocardial T1 values to blood-pool measurements.

### Image Analysis

Analysis of all MRI scans from both centers was performed at the Edinburgh Core Lab using CVI42 (Circle Cardiovascular Imaging Inc, Calgary, Canada) by a single reporter (R.J.E.) blinded to the scan time point (Data Supplement). Short-axis cine images were used to calculate ventricular volumes, mass, and function. The presence of midwall LGE was determined both qualitatively and quantitatively by 2 experienced operators (R.J.E. and M.R.D.), and its distribution recorded. LGE was quantified in a semiautomated manner using a signal intensity threshold of >3 SDs above the mean value in a region of normal myocardium.^12^ Although segments with midwall late enhancement were included in the overall T1 calculation, segments with subendocardial infarct pattern LGE were excluded. ECV fraction (ECV%) and indexed ECV (iECV: ECV%× LV end-diastolic myocardial volume normalized to body surface area) were calculated using the motion-corrected native and postcontrast T1 maps (Data Supplement). We have previously reported the reproducibility of these measures at 3T^13^ and demonstrated that iECV acts as a marker of LV decompensation in AS, correlates with the burden of diffuse fibrosis on histology, and is associated with future clinical events.^7^ Other groups have also recently used the same parameter.^14^

### Statistical Analysis

All statistical analyses were performed using GraphPad Prism version 7.0 and SPSS version 23. A 2-sided *P*<0.05 was considered statistically significant. Given heterogeneity in timing of follow-up imaging, changes in the LV remodeling variables were annualized. Annualized change was calculated as the difference between the baseline and final follow-up MRI scans, divided by the number of days in between time points and multiplied by 365. This approach assumes that progression is linear. In a sensitivity analysis, we restricted analysis of progression and reverse remodeling in those patients who had repeat imaging at the same time interval (2 years in the natural history cohort and 1 year in the AVR cohort) and examined absolute change in the LV remodeling variables.

We assessed the distribution of all continuous variables using the Shapiro–Wilk test and presented them as appropriate using mean±SD or median (interquartile range). Annualized change was assessed using a 1 sample *t* test or Wilcoxon signed-rank test where appropriate to compare with a hypothetical mean (or median) of 0. Other comparisons were made using the Kruskal–Wallis test where appropriate. We presented all categorical variables as percentages and used the χ^2^ test for comparison. Absolute change in the sensitivity analysis was analyzed using the paired *t* test or Wilcoxon-matched pairs signed-rank test. Univariate linear regression was performed on both cohorts to investigate the change in indexed LV mass (LVMi) using variables known or suspected to influence LVM change (including age, sex, history of hypertension, and valvuloarterial impedance). Multivariable linear regression analysis was then performed with change in LVMi as the dependent variable, and the same relevant clinical variables included as covariates. R.J.E. had full access to study data and is responsible for data integrity and analysis.

## Results

Repeat MRI was performed in a total of 99 patients (n=63 from United Kingdom, n=36 from Canada; Table 1), 38 underwent AVR (AVR cohort: age, 66±8 years; 76% men; peak aortic-jet velocity, 4.70±0.83 m/s) and 61 remained under medical surveillance without intervention (natural history cohort: age, 61±12 years; 66% men; peak aortic-jet velocity, 3.24±0.76 m/s).

**Table 1.**
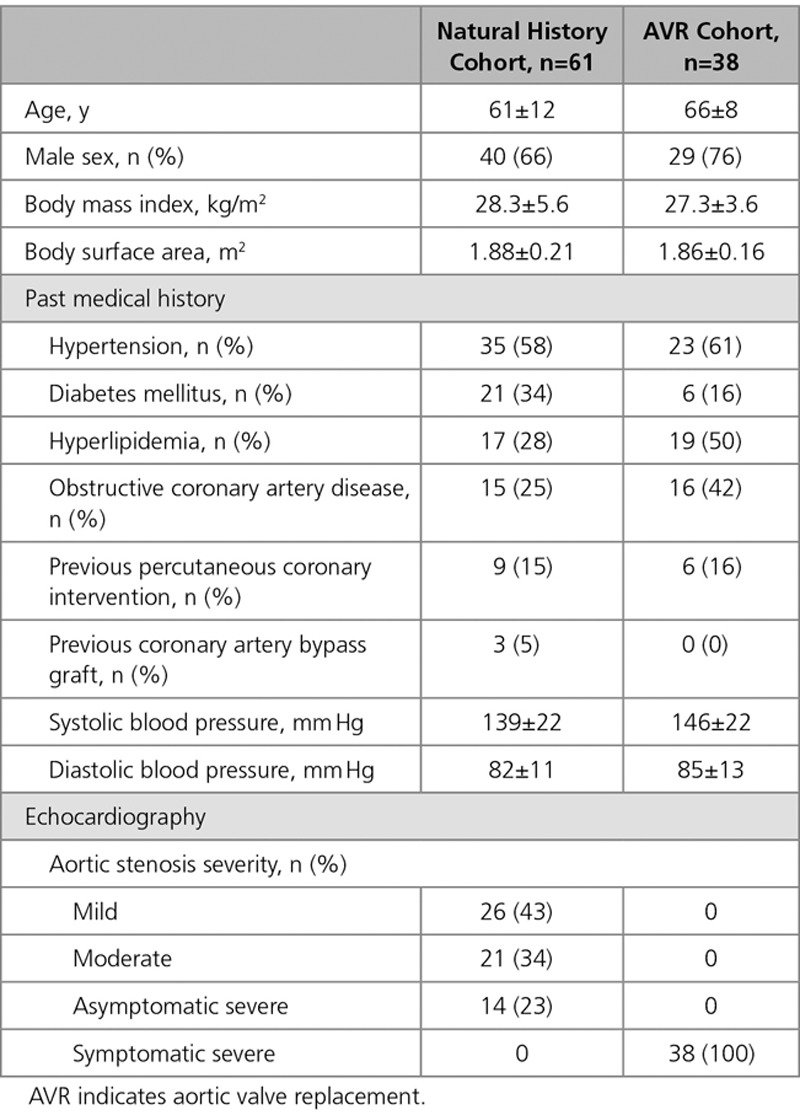
Baseline Characteristics of Patients in the Natural History and AVR Cohorts

### Natural History Cohort (LV Remodeling)

At baseline, AS was graded as mild in half of the cohort, with the remainder split between moderate (34%) and severe (23%; Table 1). No patient had symptoms attributable to valve disease. Follow-up MRI was performed at 2.1±0.7 years after baseline scan.

As expected, AS severity increased (peak aortic-jet velocity, 0.15 m/s per year [0–0.29 m/s per year]; mean gradient, 3 mm Hg/y [1–5 mm Hg/y]; aortic valve area: −0.05 cm^2^/y [−0.08 to −0.01 cm^2^/y]; *P*<0.001 for all; Table 2) with concurrent increases in both LVMi (3 g/m^2^ per year [1–5 g/m^2^ per year]; *P*<0.001) and maximum LV wall thickness (0.5 mm/y [0–1 mm/y]; *P*<0.001). These changes were accompanied by a reduction in longitudinal systolic function (−0.5 mm/y [−1.5 to 0.3 mm/y]; *P*=0.003) and an increase in LV filling pressures (E/e′, 0.6 /y [−0.4 to 1.3 /y]; *P*=0.006; Table 3). There was no significant change in LV stroke volume or ejection fraction over time (both *P*≥0.20).

**Table 2.**
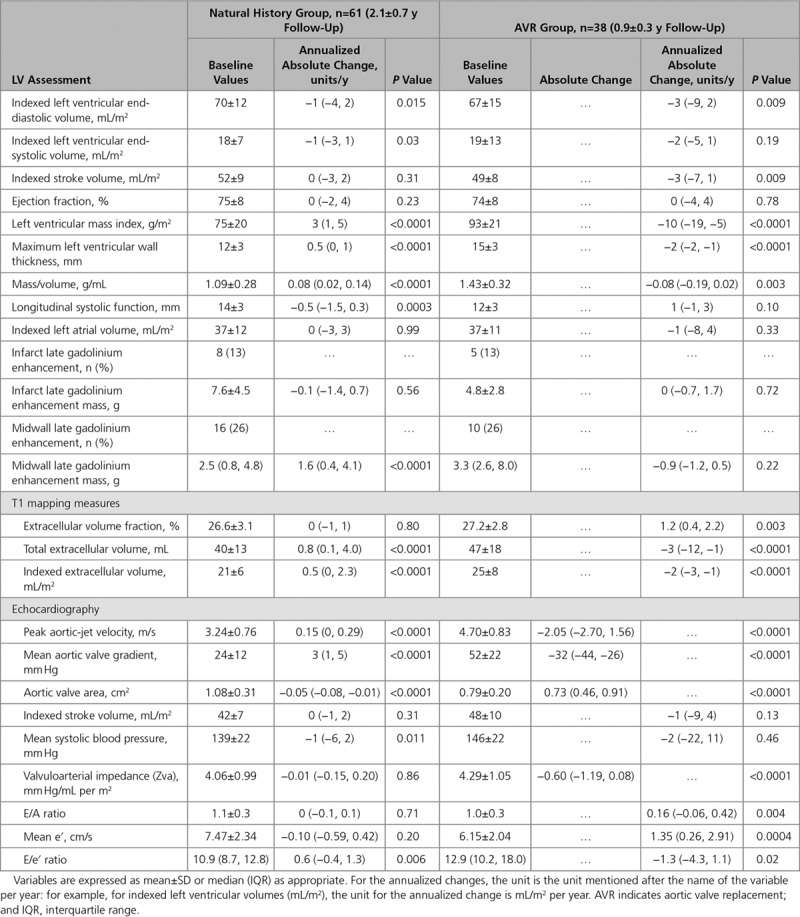
Baseline and Annualized Change in Markers of Left Ventricular Remodeling Among Patients in the Natural History and AVR Groups

**Table 3.**
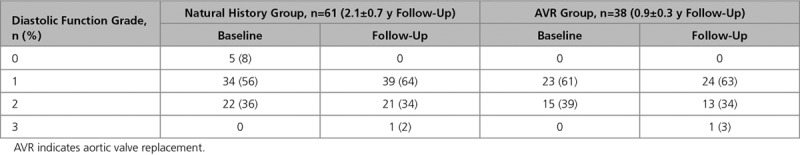
Diastolic Function Grade at Baseline and Follow-Up in the Natural History and AVR Groups

**Figure 1.**
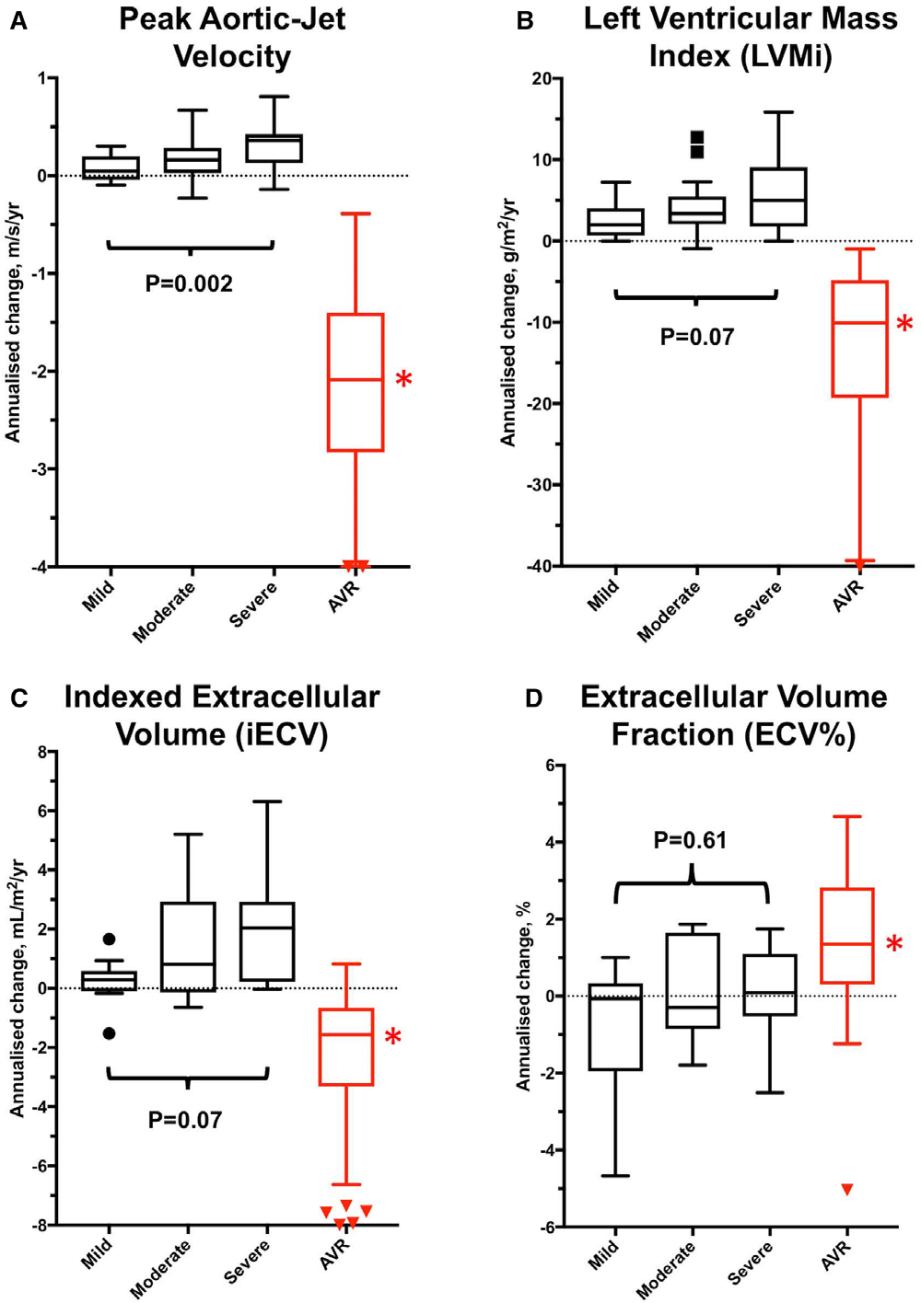
Annualized changes in aortic valve obstruction, left ventricular hypertrophy, and diffuse fibrosis in the natural history and aortic valve replacement (AVR) groups. Annualized progression in peak aortic-jet velocity (**A**), left ventricular mass (**B**), and diffuse fibrosis (indexed extracellular volume [iECV], **C**) increased in a stepwise fashion with severity of aortic stenosis. The slowest progression for each parameter was observed in patients with mild aortic stenosis and the fastest progression in those with severe stenosis. Extracellular volume fraction (ECV%) did not change (**D**), suggesting balanced progression in cellular hypertrophy and interstitial fibrosis. After AVR, there was significant regression in valve obstruction (**A**), left ventricular mass index (LVMi; **B**), and iECV (diffuse fibrosis, **C**). ECV% increased (**D**) suggesting more rapid regression in cellular hypertrophy than interstitial diffuse fibrosis (all *P*<0.005). *Significant (*P*<0.005) annualized change comparing pre- and post-AVR values for each measure.

When classified by baseline AS severity, there was a stepwise increase in the progression of both the valve stenosis severity (change in peak aortic-jet velocity: mild AS, 0.05 m/s per year [−0.03 to 0.20 m/s per year]; moderate AS, 0.16 m/s per year [−0.04 to 0.29 m/s per year]; and severe AS, 0.33 m/s per year [0.16–0.42 m/s per year]; *P*=0.002) and the hypertrophic response (change in LVMi: mild AS, 2 g/m^2^ per year [1–4 g/m^2^ per year]; moderate AS, 3 g/m^2^ per year [2–5 g/m^2^ per year]; and severe AS, 5 g/m^2^ per year [2–9 g/m^2^ per year]; *P*=0.07; Table 4; Figure 1). Indeed, a moderate correlation was observed between the rate of peak aortic-jet velocity progression and the rate of LVMi progression (*r*=0.41; *P*=0.001) with both baseline and annualized peak aortic-jet velocity change being predictors of the rate of LVMi progression on univariable analysis. Annualized change in peak aortic-jet velocity was the only independent predictor of LVMi progression on multivariable analysis (*P*=0.02; Table 5).

**Table 4.**
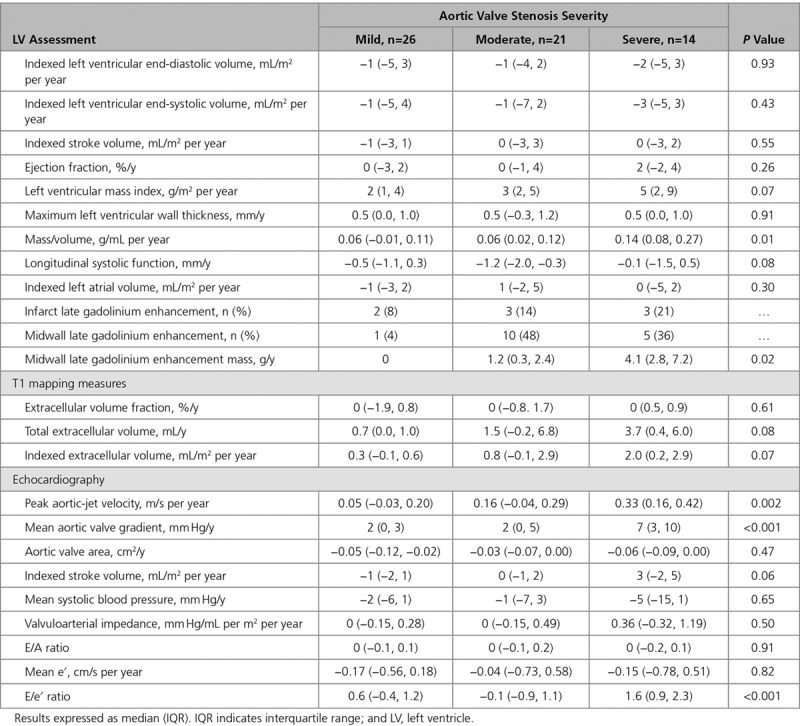
Annualized Change in Markers of Progression and Left Ventricular Remodeling According to Aortic Stenosis Severity in the Natural History Group

**Table 5.**
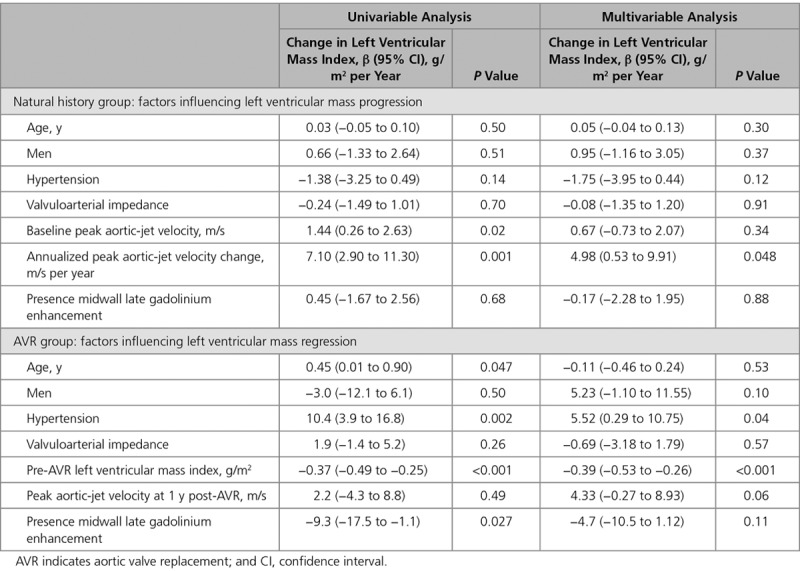
Univariable and Multivariable Linear Regression Analysis to Examine the Predictors of Annualized Progression and Regression of Left Ventricular Mass Over Time

### Myocardial Fibrosis

iECV increased over time (0.5 mL/m^2^ per year [0–2.3 mL/m^2^ per year]; *P*<0.0001; Table 2; Figure 1), with progression again appearing to increase in a stepwise manner across patients with mild (0.3 mL/m^2^ [−0.1 to 0.6 mL/m^2^]), moderate (0.8 mL/m^2^ [−0.1 to 2.9 mL/m^2^]), and severe (2.0 mL/m^2^ [0.2–2.9 mL/m^2^]) AS (*P*=0.07; Table 4). Indeed, iECV increased ≈7-fold faster in those with severe versus mild AS (*P*=0.01; Figure 1). By contrast, no progression in ECV% was observed over time either across the cohort as a whole (0% [−1% to 1%); *P*=0.80) or within severity subgroups (*P*=0.61).

**Figure 2.**
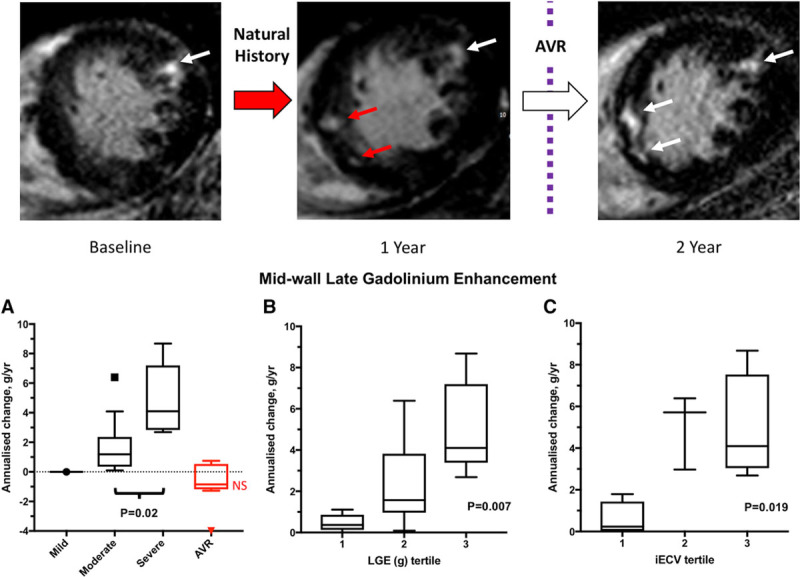
Serial magnetic resonance images in a patient with severe aortic stenosis and progression of replacement fibrosis. **Top** row, Midwall late gadolinium enhancement (LGE) is present baseline magnetic resonance imaging (MRI; white arrow, baseline image). New areas of LGE can be seen on follow-up MRI after 1 y (red arrows). The patient subsequently developed exertional breathlessness and underwent aortic valve replacement (AVR). Repeat imaging 1 y after AVR demonstrated no change in the pattern or volume of LGE. In patients with established midwall LGE, rapid accumulation of further LGE was observed with the fastest progression in those with the most severe aortic stenosis (**A**), the highest baseline burden of LGE (**B**), and the most advanced indexed extracellular volume (iECV; **C**). After AVR, there was no change in LGE burden (**A**). NS indicates no significant annualized change in AVR group compared with baseline values.

**Figure 3.**
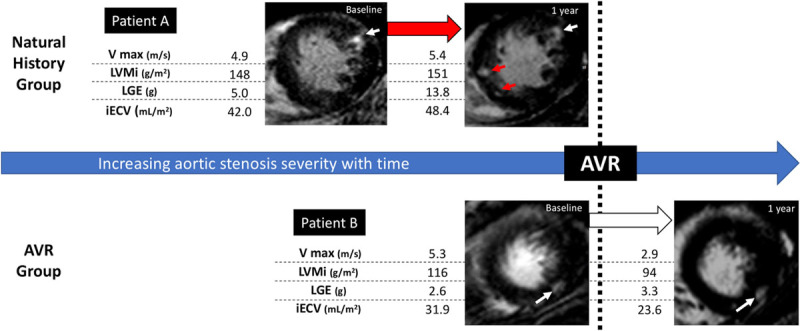
Changes in left ventricular mass (LVM), diffuse fibrosis, and replacement fibrosis in aortic stenosis before and after valve replacement. Longitudinal changes in LVM index (LVMi), diffuse fibrosis (indexed extracellular volume [iECV]), and replacement fibrosis (late gadolinium enhancement [LGE]) before and after valve replacement (AVR) are illustrated with 2 example patients (A and B). All 3 measures increase exponentially as stenosis severity increases (patient **A**, natural history cohort), and new areas of LGE are seen on follow-up imaging (red arrows). However, after AVR, cellular hypertrophy regresses more quickly than diffuse fibrosis, and replacement fibrosis seems unchanged (patient **B**, white arrows). AVR indicates aortic valve replacement; and V_max_, peak aortic-jet velocity.

Midwall LGE was present at baseline in 16 patients (26%) and progressed rapidly with time (change in LGE mass, 1.6 g/y [0.4–4.1 g/y]; *P*<0.0001; Table 2), equivalent to a relative annual progression of 78% (50%–158%). This occurred both at the sites of existing LGE and, in a quarter of patients, at remote sites with the development of new areas of midwall LGE (Figures 2 and 3). Again faster rates of progression were observed in patients with more advanced valve stenosis (*P*=0.02) and greater levels of diffuse fibrosis (*P*=0.019, by tertiles of iECV; Figure 2). Moreover, patients with the most midwall LGE at baseline demonstrated the fastest subsequent progression (tertile 1 baseline LGE, 0.3 g/y [0.1–0.9 g/y]; tertile 2, 1.6 g/y [1.0–3.8 g/y]; and tertile 3, 4.1 g/y [3.4–7.2 g/y]; *P*=0.007; Figure 2). Eight patients (13%) had a subendocardial pattern of LGE at baseline. On repeat MRI, there were no new areas of subendocardial LGE and no change in the subendocardial LGE mass (*P*=0.56; Table 2), consistent with these areas representing previous myocardial infarction.

### AVR Cohort (Reverse Remodeling)

Patients underwent AVR for a guideline-based indication 32 days (13–66 days) after baseline imaging with repeat imaging performed 0.9±0.3 years after AVR. Twenty-nine patients received a bioprosthetic AVR, and in 9 patients, a mechanical prosthesis was used. No patient underwent transcatheter valve replacement. As expected, echocardiographic assessments of aortic valve obstruction improved after surgery (change in peak aortic-jet velocity, −2.05 m/s [−2.70 to 1.56 m/s]; change in mean gradient, −32 mm Hg [−44 to −26 mm Hg]; change in aortic valve area, 0.73 cm^2^ [0.46–0.91 cm^2^]; change in valvuloarterial impedance, −0.60 [−1.19 to 0.08]; all *P*<0.0001; Table 2).

There was a 19% reduction in LVMi (−10 g/m^2^ per year [−19 to −5 g/m^2^ per year]; *P*<0.0001; Table 2) after AVR, accompanied by a corresponding reduction in maximal LV wall thickness (−2 mm/y [−2 to −1 mm/y]; *P*<0.0001). A moderate correlation was observed between the magnitude of LVM regression and the reduction in peak aortic-jet velocity after valve intervention (ρ=0.35; *P*=0.03). On multivariable regression analysis, a high pre-AVR LVMi and the absence of hypertension were both associated with greater LVM regression (Table 5) as was a lower post-AVR V_max_ although this last variable did not reach statistical significance (*P*=0.06).

Measures of LV relaxation and filling pressure improved after AVR (mean e′, 1.35 [0.26–2.91]; *P*=0.0004; E/e′, −1.3 [−4.3 to 1.1]; *P*=0.02), and there was an apparent trend toward improved longitudinal LV systolic function (1 mm/y [−1 to 3 mm/y]; *P*=0.10). No change in ejection fraction was observed (*P*=0.78) although the indexed end-diastolic LV volume did decrease modestly (−3 mL/m^2^ per year [−9 to 2 mL/m^2^ per year]; *P*=0.009).

### Myocardial Fibrosis

There was a 11% reduction in iECV on repeat imaging after AVR (−2 mL/m^2^ per year [−3 to −1 mL/m^2^ per year]; *P*<0.001; Table 2; Figure 1). In contrast, the ECV% increased (1.2% /y [0.4%–2.2% /y]; *P*=0.003; Figure 1) consistent with faster regression of LVM than diffuse fibrosis. The type of replacement valve implanted did not influence the degree of LVM (*P*=0.61) or iECV (*P*=0.97) regression.

On visual assessment, midwall LGE was present in 10 patients (26%) at baseline. No patient went on to develop new areas of LGE on repeat imaging nor did any patient with existing LGE demonstrate resolution of any established areas post-AVR (Figure 3). Quantitatively, there was no significant change in LGE mass after AVR (*P*=0.22; Table 2) even in patients rescanned after 2 years. Infarct pattern LGE was observed at baseline in 5 patients (13%). One new infarct was detected on repeat imaging, but overall no change was observed in LGE mass in these patients (*P*=0.72).

Sensitivity analysis was performed in patients who underwent repeat imaging at the same time interval (2 years in the natural history cohort, n=50; 1 year in the AVR cohort, n=27). Our findings were unchanged from those made across the cohort as a whole (Figure I and Table I in the Data Supplement).

## Discussion

This is the first study to characterize how LVH and fibrosis progress in AS and how these processes then reverse remodel after AVR. Using a multicenter multimodality imaging approach with serial echocardiography and MRI, we have demonstrated that both hypertrophy and fibrosis progress in an increasingly rapid manner as AS severity advances. Once midwall patterns of replacement fibrosis (LGE) have become established, further scarring seems to accumulate rapidly. Although LVH and diffuse fibrosis reverse after AVR, midwall LGE does not and seems to be irreversible. Given the adverse prognosis associated with midwall fibrosis burden, our data suggest prompt AVR at the first sign of midwall LGE or just before its development might improve long-term patient outcomes.

In the natural history cohort, we observed a slow and steady progression in each of the echocardiographic measures of valvular stenosis as anticipated.^15^ This valve progression was strongly influenced by baseline AS severity, with the slowest progression in patients with mild stenosis and the most rapid progression in those with severe obstruction. This was mirrored by a similar pattern of increasing LVM progression. Indeed, a moderate correlation was observed between valve stenosis progression and LVM progression, with the annualized increase in peak aortic-jet velocity, the only independent predictor of LVM progression on multivariable analysis. Consistent with this, AVR resulted in a substantial reduction in aortic valve obstruction that was accompanied by a ≈20% reduction in the LVM. Again, there was a strong correlation between the reduction in transvalvular gradient and LVM regression, with the former emerging as an independent predictor of reverse remodeling on multivariable analysis. One surprising finding was a small but significant reduction in stroke volume after AVR. This may relate to accompanying reductions in LV end-diastolic volume but requires further study.

What about myocardial fibrosis? MRI is the only noninvasive imaging technique capable of assessing both diffuse interstitial (T1 mapping techniques) and replacement fibrosis (LGE). T1 mapping provides multiple different measurements that demonstrate close agreement with collagen volume fraction on histology and therefore act as surrogates of interstitial myocardial fibrosis.^7,16^ We here investigated the ECV% and iECV because of the advantages these measures hold when comparing values acquired in a multicenter setting on different scanners and at different field strengths. Although ECV% gives an indication of the proportion of the myocardium made up of fibrosis, iECV is a surrogate of the total fibrosis burden in the LV. Together these 2 measures can provide unique insights into how the extracellular and intracellular compartments of the myocardium change in AS and in response to AVR. Like peak aortic-jet velocity and LVMi, the iECV increased with time suggesting progressive expansion of the extracellular compartment and diffuse interstitial fibrosis. Once again, this progression appeared to occur quickest in those with the most advanced valvular stenosis. By comparison, ECV% did not demonstrate any evidence of progression, suggesting balanced increases in the size of the cellular and extracellular compartments as LV remodeling advances.

After AVR, reductions in iECV were observed similar to those observed in peak aortic-jet velocity and LVM, confirming that diffuse interstitial fibrosis is indeed reversible. However, the accompanying rise in ECV% suggests that regression in cellular hypertrophy occurs faster and to a greater degree than this reduction in diffuse fibrosis. These novel imaging findings are in keeping with historical data from myocardial biopsies performed after AVR showing an initial increase of percentage interstitial fibrosis on histology at 18 months.^17^

Midwall LGE represents a more advanced stage of focal replacement fibrosis^18^ in the myocardium and has been described in numerous AS populations.^4,6,19^ Midwall LGE is a marker of LV decompensation demonstrating a close association with myocardial injury, LV diastolic function, LV systolic function, and exercise capacity.^7^ Moreover, multiple different studies from multiple centers have confirmed midwall LGE as a powerful prognostic marker of long-term all-cause and cardiovascular mortality.^4–7^ Most of these adverse events occur after AVR,^20^ and there seems to be a proportionate relationship: the more myocardial LGE, the worse the clinical outcomes.^4,5^

For the first time, we have demonstrated that the burden of midwall LGE increases while asymptomatic patients are being monitored in the clinic. Indeed, once midwall LGE has become established, then further accumulation of such scarring is relatively rapid, increasing on average by 75% each year especially in patients with a high baseline fibrosis burden. Importantly, we go on to demonstrate that although this progressive scarring is arrested by AVR, it does not reverse even out to 2 years after AVR. This is consistent with smaller short-term studies and implies that the scar that patients develop while waiting for surgery remains with them for the rest of their life, contributing to their poorer long-term prognosis. These findings could have important clinical implications for optimizing patient care and the timing of AVR. For example, based on our data, prompt AVR could be undertaken when midwall LGE is first identified to prevent the accumulation of further scarring and to improve long-term patient outcomes. This strategy requires prospective confirmation and is currently being tested in the EVOLVED (Early Valve Replacement guided by Biomarkers of Left Ventricular Decompensation in Asymptomatic Patients with Severe Aortic Stenosis) randomized controlled trial (NCT03094143).

Our study does have some limitations. Given the heterogeneity in the timing of follow-up imaging, we used annualized change for our primary analysis. This assumes linear progression or regression of variables which may not be the case. In the sensitivity analysis, we repeated our analysis of the data using absolute change in the subgroup of patients who underwent repeat imaging after the same time interval (2 years in the natural history cohort [n=50] and 1 year in the AVR cohort [n=27]). Our results were consistent with the annualized analysis. Further studies are still required to investigate how LV remodeling and reverse remodeling progress over multiple time points in individual patients. The ECV measurements (ECV%, iECV) reflect the size of the extracellular compartment and therefore potentially represent multiple different factors, including the intravascular space and myocardial infiltration. However, in patients with AS (and in the absence of associated cardiac amyloidosis), there is a close association between these ECV measurements and histological markers of interstitial fibrosis, confirming that they provide a useful surrogate measure of interstitial fibrosis, as here presented.

## Conclusions

We have used echocardiography and MRI to characterize the structural changes in the myocardium that occur in patients with AS both during routine surveillance and after AVR. In patients with AS, cellular hypertrophy and diffuse interstitial fibrosis increase in a balanced and exponential manner before reversing at different rates after AVR. Once established, midwall replacement fibrosis accumulates rapidly but seems irreversible after AVR. The myocardial scar burden that patients develop while waiting for surgery, therefore, persists into the long term along with prognostic implications that this entails. Prompt valve replacement as soon as midwall fibrosis develops holds promise in improving clinical outcomes in patients with AS.

## Sources of Funding

The work was supported by the British Heart Foundation (CH/09/002/26360 to Dr Newby RE/13/3/30183 to Dr Prasad FS/14/78/31020 to Dr Dweck), the Wellcome Trust (WT103782AIA to Dr Newby), the Sir Jules Thorn Charitable Trust (15/JTA to Dr Dweck), the Québec Heart and Lung Institute Foundation, and Canadian Institutes of Health Research (FDN-143225 and MOP-114997 to Drs Pibarot and Clavel).

## Disclosures

None.

## Supplementary Material
