# Supplementary material for: Progression of Hypertrophy and Myocardial Fibrosis in Aortic Stenosis: A Multicenter Cardiac Magnetic Resonance Study
Source: Circ Cardiovasc Imaging. 2018 Jun 18;11(6):e007451. doi: 10.1161/CIRCIMAGING.117.007451 (PMC6023592; doi:10.1161/CIRCIMAGING.117.007451)

SUPPLEMENTAL MATERIAL

Progression of Hypertrophy and Myocardial Fibrosis in Aortic Stenosis: A Multicenter Cardiac Magnetic Resonance Study

Everett, Myocardial fibrosis progression in aortic stenosis

Russell J Everett*, Lionel Tastet*, Marie-Annick Clavel, Calvin WL Chin, Romain Capoulade, Vassilios S Vassiliou, Jacek Kwiecinski, Miquel Gomez, Edwin JR van Beek, Audrey C White, Sanjay K Prasad, Eric Larose, Christopher Tuck, Scott Semple, David E Newby, Philippe Pibarot^, Marc R Dweck^

*Joint contribution as first author
^Joint contribution as senior author

CORRESPONDING AUTHOR
Russell Everett, MD
Chancellor's Building
University of Edinburgh
49 Little France Crescent
EH16 4SB

Email: Russell.everett@ed.ac.uk
Telephone: 01312426361
Fax: 01312426379

Methods

Echocardiography

A comprehensive transthoracic echocardiographic assessment was performed in all patients (Edinburgh: iE33, Philips Medical Systems, The Netherlands. Quebec: iE33 or EPIQ, Philips Healthcare, Ontario, Canada) by dedicated research ultrasonographers. Careful attention was given in the assessment of aortic stenosis severity. The left ventricular (LV) outflow tract diameter was measured in the parasternal long-axis view, at the insertion of the aortic cusps from the inner edge of the septal endocardium to the inner edge of the anterior mitral leaflet in mid-systole. Left ventricular outflow tract velocity-time integral was measured in the apical 5-chamber view using pulsed-wave Doppler just proximal to the aortic valve, with care taken to obtain a laminar spectral tracing. The peak aortic jet velocity and mean transvalvular gradient were derived from the aortic valve velocity-time integral, using continuous-wave Doppler. The highest aortic jet velocity and mean transvalvular gradient were determined in multiple acoustic windows using both standard S51 and D2cwc probes (Philips Medical Systems, Best, the Netherlands). The mean of 3 readings (5 if the patient had atrial fibrillation) was recorded. Aortic valve area was calculated using the continuity equation. The severity of aortic stenosis was assessed and classified according to the European Association of Echocardiography/American Society of Echocardiography guidelines.[1]

Trans-mitral early (E) and late diastolic velocities, as well as, deceleration time of early filling velocity were measured at the tips of the mitral valve leaflets using pulsed-wave Doppler. The mean early diastolic velocities of the medial and lateral mitral annulus (e’) were measured using pulsed-wave tissue Doppler imaging. Diastolic function was assessed as recommended in recent guidelines.[2]

Magnetic resonance imaging

Magnetic resonance imaging was performed using both 1.5 and 3T scanners (Edinburgh: MAGNETOM Verio, Siemens AG, Erlangen, Germany; Quebec: ACHIEVA and INGENIA, Philips Healthcare, Best, the Netherlands or, Erlangen, Germany). Repeat imaging was performed using the same standardized protocols at each site. Short-axis cine images extending from the mitral valve to the left ventricular apex were obtained using a balanced steady-state free precession sequence (Edinburgh: 8-mm parallel slices with 2-mm spacing; temporal resolution ≤45ms. Quebec: 8 mm parallel slices with no gap). Typical parameters at 1.5T were FOV 380 mm, TR/TE 3.2/1.6 ms, flip angle 60º and NEX of 1, in-plane spatial resolution of 1.6 x 2 mm. Equivalent acquisition parameters at 3T were FOV 380 mm, TR/TE 2.8/1.3 ms, flip angle 45º, and NEX of 1, in-plane spatial resolution of 1.7 mm x 2 mm, 7-mm slice thickness, 0-mm gap.

Focal replacement and diffuse interstitial myocardial fibrosis was assessed in all patients using late gadolinium enhancement (LGE) and myocardial T1 mapping, respectively. Late gadolinium enhancement was performed 15 min following gadobutrol (Gadovist, Bayer Pharma AG, Germany, 0.1 mmol/kg [Edinburgh], 0.2 mmol/kg [Quebec]) using an inversion-recovery fast gradient-echo sequence performed in two phase-encoding directions to differentiate true late enhancement from artefact. The LGE imaging parameters at 1.5T were FOV 350 mm, TR/TE 4.5/1.3 ms, flip angle 15 º, 8-mm slice thickness, in-plane resolution of 1.9 mm x 3.1 mm with an inversion time of 200 to 300 ms adjusted to null normal myocardium following gadolinium contrast administration. Equivalent acquisition parameters at 3T were FOV 350 mm, TR/TE 6.1/3 ms, flip angle 25 º, 8 mm slice thickness, in-plane resolution of 1.6 mm x 2 mm. The inversion time was optimized to achieve satisfactory nulling of the myocardium.

Diffuse myocardial fibrosis was assessed using Modified Look-Locker Inversion-recovery with built-in motion correction. A heart beat acquisition scheme of 3(3)-3(3)-5 was used in Edinburgh (flip angle

35°; minimum TI 100 ms; TI increment of 80 ms; time delay of 150 ms)[3,4,5] whilst an acquisition scheme of 5(3)-3 was used in Quebec (with a post-contrast acquisition scheme of 4(1)3(1)2 used in patients scanned at 3T).[6] A gradient echo field map and associated shim were performed to minimize off-frequency artefact.

Image analysis

Ventricular volumes, mass and function were quantified using dedicated software (CVI42 (Circle Cardiovascular Imaging Inc., Calgary, Canada) by a single reporter (RJE) blinded to the scan time-point. Basal ventricular slices were included if >50% of the LV blood pool was surrounded by myocardium. Papillary muscles and minor trabeculations were included in the left ventricular mass measurements and excluded from the intracavity volume measurements as per Society for Cardiovascular Magnetic Resonance guidelines.[7]

The left ventricular wall thickness was measured in each of the 16 myocardial segments (excluding the LV apex) and the maximum value recorded. Left ventricular longitudinal function was determined by measuring the difference in the distance between the mitral valve plane and the epicardial left ventricular apex in end-systole and end-diastole. The final value was calculated as the mean value of the recorded measurements in both 4-chamber and 2-chamber views. Left atrial volume was calculated using the bi-plane area-length method by tracing the endocardial LA contour in end-ventricular systole in both 2 and 4 chamber long-axis views.

The presence of mid-wall myocardial fibrosis was determined qualitatively by two independent and experienced operators (MRD and RJE). The distribution of mid-wall fibrosis was described according to the standard 17-segment model recommended by the American College of Cardiology/American Heart Association.[8] LGE was quantified in a semi-automated manner using a signal intensity threshold of >3 standard deviations above the mean value in a region of normal myocardium.[9] Areas of inversion

artefact, infarct pattern LGE or signal contamination by epicardial fat or blood pool were manually excluded. Sub-endocardial LGE was also identified and quantified using the same analysis technique.

T1 mapping analysis was performed using CVI42 (Circle Cardiovascular Imaging Inc., Calgary, Canada). Endocardial and epicardial contours were manually contoured on the native motion-corrected myocardial T1 maps with manual offsetting of the contours to avoid partial volume effects. The right ventricular insertion points were identified leading to automatic segmentation of the basal and mid-ventricular slices. No analysis was performed on the apical myocardial segments as these are most susceptible to partial volume effects. These contours were subsequently copied onto corresponding 20-minute post-contrast maps with minor adjustments made to avoid partial volume effects and artifact. Segments demonstrating mid-wall late enhancement were included in the overall T1 analysis whereas those containing infarct pattern LGE were excluded as per recent post-processing guidelines.[10] The extracellular volume fraction (ECV%) was calculated according to: ECV% = partition coefficient x [1-haematocrit], where partition coefficient = $[\Delta R1_{myocardium}/\Delta R1_{blood\text{-}pool}]$ and $\Delta R1$ = (1/post-contrast T1-1/pre-contrast T1). This was calculated based on the average of the values obtained from the basal and mid ventricular segments. Hematocrit was sampled at the time of cardiovascular magnetic resonance imaging. The indexed extracellular volume (iECV) in each patient was derived using the following: ECV% x left ventricular end-diastolic myocardial volume indexed to body surface area (using the Dubois formula), where left ventricular myocardial volume = left ventricular mass /1.05 g/mL.[11,12]

References

1. Baumgartner H, Hung J, Bermejo J, Chambers JB, Edvardsen T, Goldstein S, Lancellotti P, LeFevre M, Miller F, Otto CM. Recommendations on the Echocardiographic Assessment of Aortic Valve Stenosis: A Focused Update from the European Association of Cardiovascular Imaging and the American Society of Echocardiography. *J Am Soc Echocardiogr*. 2017;30:372–392.

2. Nagueh SF, Smiseth OA, Appleton CP, Byrd BF, Dokainish H, Edvardsen T, Flachskampf FA, Gillebert TC, Klein AL, Lancellotti P, Marino P, Oh JK, Alexandru Popescu B, Waggoner AD, Houston, Texas; Oslo, Norway; Phoenix, Arizona; Nashville, Tennessee; Hamilton, Ontario, Canada; Uppsala, Sweden; Ghent and Liège, Belgium; Cleveland, Ohio; Novara, Italy; Rochester, Minnesota; Bucharest, Romania; and St. Louis, Missouri. Recommendations for the Evaluation of Left Ventricular Diastolic Function by Echocardiography: An Update from the American Society of Echocardiography and the European Association of Cardiovascular Imaging. *Eur Heart J - Cardiovasc Imaging*. 2016;17:1321–1360.

3. Messroghli DR, Radjenovic A, Kozerke S, Higgins DM, Sivananthan MU, Ridgway JP. Modified Look-Locker inversion recovery (MOLLI) for high-resolution T1 mapping of the heart. *Magn Reson Med*. 2004;52:141–146.

4. Messroghli DR, Greiser A, Fröhlich M, Fr o hlich M, Dietz R, Schulz-Menger J. Optimization and validation of a fully-integrated pulse sequence for modified look-locker inversion-recovery (MOLLI) T1 mapping of the heart. *J Magn Reson Imaging*. 2007;26:1081–1086.

5. Xue H, Shah S, Greiser A, Guetter C, Littmann A, Jolly M-P, Arai AE, Zuehlsdorff S, Guehring J, Kellman P. Motion correction for myocardial T1 mapping using image registration with synthetic image estimation. *Magn Reson Med*. 2012;67:1644–1655.

6. Kellman P, Hansen MS. T1-mapping in the heart: accuracy and precision. *J Cardiovasc Magn Reson* 2014;16:2.

7. Schulz-Menger J, Bluemke DA, Bremerich J, Flamm SD, Fogel MA, Friedrich MG, Kim RJ, Knobelsdorff-Brenkenhoff von F, Kramer CM, Pennell DJ, Plein S, Nagel E. Standardized image interpretation and post processing in cardiovascular magnetic resonance: Society for Cardiovascular Magnetic Resonance (SCMR) board of trustees task force on standardized post processing. *J Cardiovasc Magn Reson*. 2013;15:35.

8. Cerqueira MD, Cerqueira M. Standardized Myocardial Segmentation and Nomenclature for Tomographic Imaging of the Heart: A Statement for Healthcare Professionals From the Cardiac Imaging Committee of the Council on Clinical Cardiology of the American Heart Association. *Circulation*. 2002;105:539–542.

9. Mikami Y, Kolman L, Joncas SX, Stirrat J, Scholl D, Rajchl M, Lydell CP, Weeks SG, Howarth AG, White JA. Accuracy and reproducibility of semi-automated late gadolinium enhancement quantification techniques in patients with hypertrophic cardiomyopathy. *J Cardiovasc Magn Reson*. 2014;16:85.

10. Messroghli DR, Moon JC, Ferreira VM, Grosse-Wortmann L, He T, Kellman P, Mascherbauer J, Nezafat R, Salerno M, Schelbert EB, Taylor AJ, Thompson R, Ugander M, van Heeswijk RB, Friedrich MG. Clinical recommendations for cardiovascular magnetic resonance mapping of

T1, T2, T2* and extracellular volume: A consensus statement by the Society for Cardiovascular Magnetic Resonance (SCMR) endorsed by the European Association for Cardiovascular Imaging (EACVI). *J Cardiovasc Magn Reson*. 2017;19:75.

11. Flett AS, Sado DM, Quarta G, Mirabel M, Pellerin D, Herrey AS, Hausenloy DJ, Ariti C, Yap J, Kolvekar S, Taylor AM, Moon JC. Diffuse myocardial fibrosis in severe aortic stenosis: an equilibrium contrast cardiovascular magnetic resonance study. *Eur Heart J - Cardiovasc Imaging*. 2012;13:jes102–826.

12. Doltra A, Messroghli D, Stawowy P, Hassel J-H, Gebker R, Leppänen O, Gräfe M, Schneeweis C, Schnackenburg B, Fleck E, Kelle S. Potential reduction of interstitial myocardial fibrosis with renal denervation. *J Am Heart Assoc*. 2014;3:e001353–e001353.

Supplemental Table 1: Baseline and Absolute Change in Markers of Left Ventricular Remodeling at 2 Years in the Natural History Group and 1 Year in the AVR Group.

	NATURAL HISTORY GROUP N = 50			AVR GROUP N=27		
**LV assessment**	**Baseline values**	**2-year absolute change**	**P value**	**Baseline values**	**1-year absolute change**	**P value**
Indexed left ventricular-end diastolic volume, mL/m²	69±11	-2 [-8, 5]	0.058	68±16	-4 [-18, 6]	0.047
Indexed left ventricular-end systolic volume, mL/m²	15 [13, 22]	-2 [-5, 3]	0.07	19 [13, 25]	-3 [-8, 4]	0.22
Indexed stroke volume, mL/m²	52±9	-1 [-6, 4]	0.47	48±9	-3 [-14, 4]	0.08
Ejection fraction, %	75±9	0 [-4, 8]	0.15	73±9	1 [-5, 7]	0.77
Left ventricular mass index, g/m²	72±17	5 [2, 12]	<0.0001	97±23	-18 [-26, -7]	<0.0001
Maximum left ventricular wall thickness, mm	12±3	1 [0, 2]	<0.0001	15±3	-2 [-4, -2]	<0.0001
Mass/volume	1.06±0.28	0.15 [0.02, 0.29]	<0.0001	1.50±0.35	-0.17 [-0.34, -0.07]	0.02
Longitudinal systolic function, mm	14±3	-1 [-3, 0.5]	0.0005	11±3	3 [-1, 5]	0.003
Indexed left atrial volume, mL/m²	37±11	-1 [-7, 5]	0.79	36±11	-12 [-26, 0]	0.08
Infarct late gadolinium enhancement, n (%)	4 (8)	4 (8)	-	3 (11)	4 (15)	-
Infarct late gadolinium enhancement mass, g	8.2±4.9	0.6 [-1.8, 1.4]	0.94	4.8±2.8	0 [-0.8, 1.7]	0.72
Mid-wall late gadolinium enhancement, n (%)	10 (20)	10 (20)	-	10 (37)	10 (37)	-
Mid-wall late gadolinium enhancement mass, g	1.2 [0.5, 4.5]	2.6 [0.7, 6.0]	0.002	3.6 [2.6, 9.6]	-1 [-1.4, 0]	0.14
**T1 mapping measures**						
Extracellular volume fraction, %	26.6±3.4	0.1 [-1.0, 1.5]	0.89	26.9±2.5	1.4 [0.8, 4.0]	<0.0001
Total extracellular volume, mL	38±11	2 [4, 1]	0.001	50±20	-4 [-12, -1]	<0.0001
Indexed extracellular volume, mL/m²	20±5	1 [0, 4]	0.001	27±9	-2 [-7, -1]	<0.0001
**Echocardiography**						
Peak aortic-jet velocity, m/s	3.13±0.73	0.25 [-0.05, 0.51]	<0.0001	4.73±0.91	-2.15 [-2.78, -1.60]	<0.0001
Mean aortic valve gradient, mmHg	23±12	4 [1, 9]	<0.0001	54±25	-32 [-48, -26]	<0.0001
Aortic valve area, cm²	1.12±0.31	-0.09 [-0.16, -0.02]	<0.0001	0.79±0.22	0.70 [0.49, 1.06]	<0.0001
Indexed stroke volume, mL/m²	42±7	-1 [-3, 3]	0.73	47±10	-2 [-8, 3]	0.028
Mean systolic blood pressure, mmHg	138±20	-3 [-12, 5]	0.06	143±21	-4 [-17, 12]	0.15
Valvuloarterial impedance (Zva), mmHg/mL/m²	3.99±0.97	0.02 [-0.28, 0.44]	0.26	4.33±1.14	-0.56 [-1.39, -0.05]	0.0004
Mean e', cm/s	7.58±2.07	-0.10 [-1.17, 0.96]	0.33	6.05±2.01	1.38 [-0.12, 3.37]	0.003
E/e' ratio	10.9 [8.7, 11.9]	1.2 [-0.8, 2.5]	0.019	12.9 [10.1, 18.0]	-1 [-5, 3]	0.56

Supplemental Figure 1: Absolute Changes in Aortic Valve Obstruction, Left Ventricular Hypertrophy and Diffuse Fibrosis in the Natural History and AVR Groups.

Absolute change in measures was assessed at the time-point with the majority of patient follow-up in the Natural History (2 years, N=50) and the AVR (1 year, N=27) groups. As in the annualised change analysis, there is an increase in rate of progression of aortic-jet velocity, LVMi and iECV with increasing AS severity in the Natural History group. However ECV fraction does not change at 2 years. Following AVR, there is regression of both LVMi and iECV, and again consistent with the annualised change analysis, we see an increase in ECV fraction, suggesting that cellular hypertrophy regresses more quickly than diffuse fibrosis.

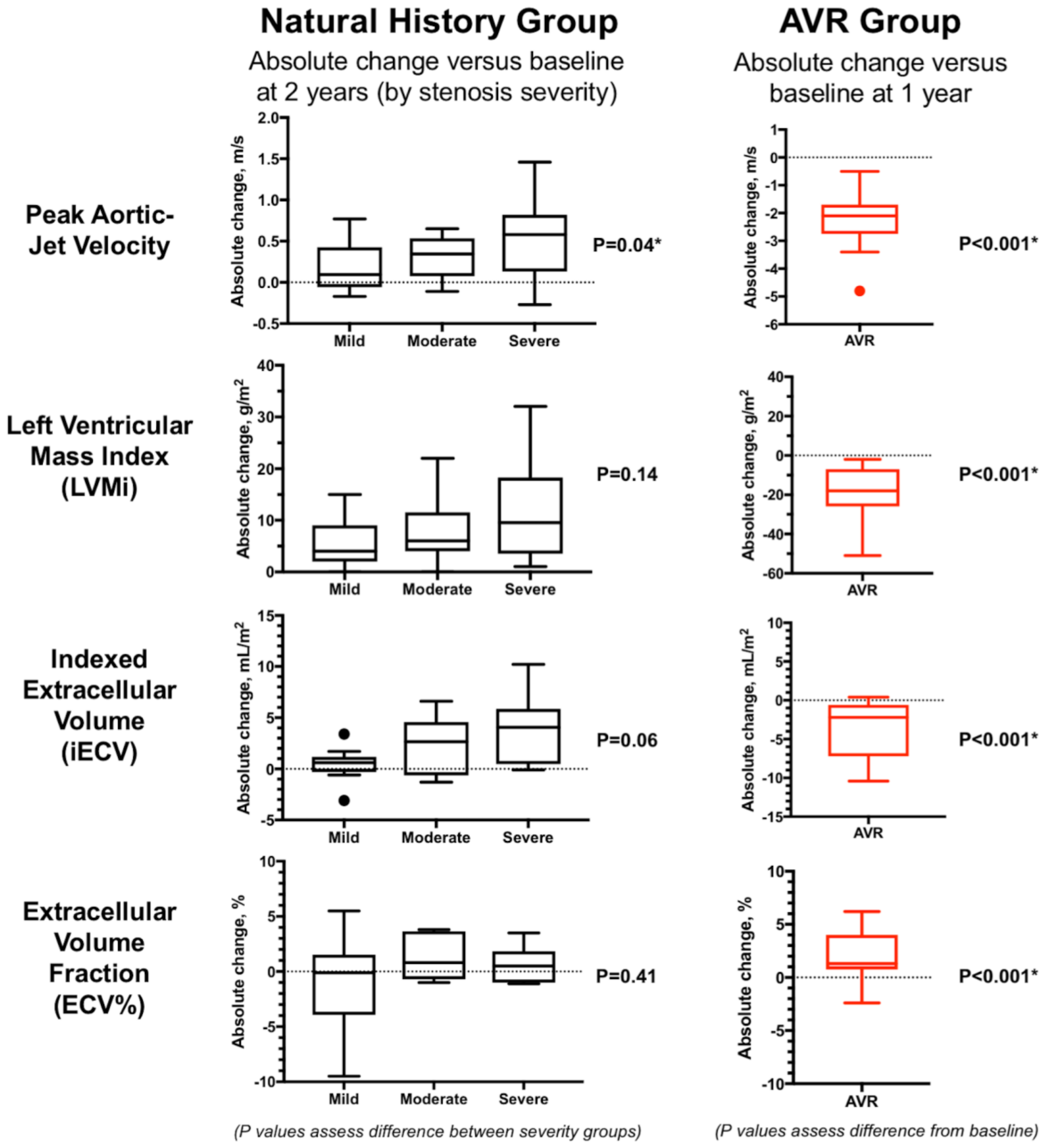

Supplement: Supplementary file 1 [file hci-11-e007451-s001.pdf]
